# A Retrospective Analysis of the Impact of Metastasectomy on Prognostic Survival According to Metastatic Organs in Patients With Metastatic Renal Cell Carcinoma

**DOI:** 10.3389/fonc.2019.00413

**Published:** 2019-05-22

**Authors:** Sung Han Kim, Weon Seo Park, Boram Park, Sahyun Pak, Jinsoo Chung

**Affiliations:** ^1^Department of Urology, Center for Urologic Cancer, Research Institute and Hospital of National Cancer Center, Goyang-si, South Korea; ^2^Department of Pathology, Hospital of National Cancer Center, Center for Urologic Cancer, Goyang-si, South Korea; ^3^Biostatistics Collaboration Team, Research Core Center, Research Institute of National Cancer Center, Goyang-si, South Korea

**Keywords:** metastasis, renal cell carcinoma, metastasectomy, organs, survival

## Abstract

This study evaluated the effects of metastasectomy on overall survival (OS) and progression-free survival (PFS) in metastatic renal cell carcinoma (mRCC) according to metastatic organs. The medical records (2005–2017) of 273 patients with mRCC were analyzed retrospectively to evaluate OS and PFS according to metastatic organs and their metastasectomy states. The Cox proportional hazard model was used to determine the prognostic significance of metastasectomy. The Kaplan-Meier curve and log-rank test were used to compare groups with different modalities and metastatic organs at a statistical significance of *p* < 0.05. The overall median age was 57 years; 175 (64.3%) and 83 (30.4%) patients received cytoreductive nephrectomy and metastasectomy, respectively. The metastasectomy group was significantly younger and had a lower clinical T stage with significantly better PFS/OS (20.2/32.0 vs. 9.7/12.8 months) than that in the non-metastasectomy group (*N* = 190, *p* < 0.05). Liver with lung metastases were the worst metastatic combination for survivals in which liver metastasis was the only significant unfavorable risk factor for both PFS (HR 1.67) and OS (HR 1.74) (*p* < 0.05). Multivariable analysis confirmed that metastasectomy was a significant favorable risk factor for PFS (HR 0.70) and OS (HR 0.56) (*p* < 0.05) along with non-clear cell type (HR 0.61 for PFS), whereas the nuclear grade and poor Heng risk group were unfavorable risk factors (HR > 2.0) for both PFS and OS (*p* < 0.05). Metastasectomy and the affected metastatic organs significantly influenced prognostic survival in mRCC.

## Introduction

Metastasis is present in about 25–35% of cases of newly diagnosed renal cell carcinoma (RCC); moreover, metastasis occurs in about 20–40% of localized primary RCC following surgical resection. The estimated 5-year overall survival (OS) for metastatic RCC (mRCC) is <20% (1, 2). Because there is not yet an effective systemic therapy for mRCC, in the cytokine era, surgical reduction of tumor burden, either by cytoreductive nephrectomy or metastasectomy of mRCC, significantly improves prognostic survival (2–4). However, in the current targeted therapy era, the benefit of surgical resection of mRCC has not been clearly identified; furthermore, targeted agents have resulted in a modest tumor reduction of 20–30%, with improved progression-free survival (PFS) and OS of about 7–9 months compared to that of the past cytokine era (5, 6).

A recent CARMENA (NCT00930033) trial demonstrated a decreasing role of cytoreductive nephrectomy for mRCC compared to that of sunitinib alone (7). Although this trial showed a targeted agent with therapeutic role comparable to that of cytoreductive nephrectomy, there may be a beneficiary role in favorable and intermediate-risk groups because the enrolled patients of the CARMENA trial included about 40% poor-risk patients. Furthermore, the advent of multiple targeted and combination therapies for mRCC has created question regarding the role of surgery for both primary RCC and metastatic tumors in terms of prognostic survival.

In association with a greater potential for better survival outcomes, including cure rates, with multiple new, targeted agents, there is also a question concerning the surgical role of metastasectomy, based on the affected metastatic organs in mRCC. This has elicited debates in the current era of targeted therapy. Nonetheless, no randomized clinical trials have been conducted to determine the role of metastasectomy; this is because the majority of studies took place in or overlapped with the immunotherapy era, in which it is difficult to isolate and analyze the heterogeneity of timing and the type of target agents used. Previous multiple retrospective studies and the European Association of Urology (EAU) RCC guidelines, which are based on a panel of systematic reviews, report a positive benefit of metastasectomy for survival rate, including PFS and OS, as well as delayed systemic therapy and exposure to adverse events (8, 9). A combination of targeted agents in conjunction with metastasectomy may result in better prognostic outcomes than those of metastasectomy alone and its complete resectability for mRCC (8–10).

However, there are few data regarding the prognostic outcomes of combined metastatic tumor burden based on the metastatic organs, along with multiple known predisposing clinicopathological parameters, in patients with mRCC following metastasectomy. Therefore, this study aimed to compare the effects of metastasectomy to those of non-metastasectomy regarding OS and PFS in synchronous mRCC according to metastatic organs and multiple clinicopathological parameters; this study also aimed to determine the prognostic risk factors for patients who underwent either metastasectomy or non-metastasectomy for mRCC.

## Materials and Methods

### Ethics Statement

Following approval of this retrospective study by the Institutional Review Board of the National Cancer Center (IRB No. NCC 2015-0212), the IRB approved exemption from the written consent procedure. All study protocols were performed in accordance with the tenets of the ethical guidelines and regulations of the “World Medical Association Declaration of Helsinki-Ethical Principles for Medical Research Involving Human Subjects.”

### Patient Criteria

The medical records (2005–17) of 273 patients with mRCC were obtained from a previously prospectively registered RCC registry database of the institution (11, 12). Baseline information regarding age, sex, nephrectomy, metastasectomy, TNM stage, histology, Fuhrman nuclear grade, and survival outcomes were included. Prognostic outcomes were analyzed retrospectively according to metastatic organs and the therapeutic modalities including cytoreductive nephrectomy (175, 64.3%), metastasectomy (83, 30.4%), radiation therapy (114, 41.8%), and embolization (38, 14.0%) (Supplementary Figure 1). Exclusion criteria included a non-complete history of survival outcomes in the National Cancer Registry Database, non-complete surgical records concerning metastasectomy or nephrectomy, or age <19 years.

### Metastasectomy With or Without Cytoreductive Nephrectomy

Cytoreductive nephrectomy was performed by a single urologist (JC) assisted by a second urologist (SHK) in cooperation with other hepatic, colorectal, thoracic, and neurosurgeons from different departments involved with the metastasectomy. Complete metastasectomy was proposed in patients with good performance status and completely resectable lesions to remove all the radiologically evident tumors on imaging studies, preferably if the tumor was localized in a single organ or, when multiple organs exhibited metastasis, whether the patient could be considered as a suitable candidate for multiple metastasectomies or complementary medical treatment. The radiation or embolization for metastatic lesions was performed when the metastasectomy might not be completely removed all the radiological evident metastases such as brain and liver metastases.

### Therapeutic Regimen of Targeted Agents and Risk Evaluations

The International Metastatic Renal Cell Carcinoma Database Consortium risk criteria (also known as the Heng criteria) and Memorial Sloan-Kettering Cancer Center (MSKCC) risk criteria (13) for prognostic risk stratification, the Response Evaluation Criteria in Solid Tumors v1.1 for therapeutic responsive evaluation to systemic therapy (14), and the Fuhrman nuclear grade and TNM stages for pathological RCC evaluation were used.

The choice of targeted agents was at the discretion of the treating urologist (JC) according to each patient's pathology and coverage by the Korean National Health Insurance System, as described previously (15). All TTs were administered either orally or intravenously with the recommended regimen in the National Comprehensive Cancer Network guidelines, version 2.2016 (available at www.nccn.org/patients for patients) and Korean national Insurance guideline. First-line TT comprised sunitinib, sorafenib, pazopanib, or temsirolimus; sequential TT included sunitinib, sorafenib, pazopanib, temsirolimus, bevacizumab, everolimus, or axitinib. The systemic therapy regimens were changed when the progression was detected on the follow-up imaging studies according to the RECIST criteria v1.1. The follow-up protocols for each target agents were suggested in previous papers (11, 12).

### Statistical Analysis

Baseline characteristics are presented as frequency (percentage) for categorical variables and median (range) for continuous variables. Differences in distributions were compared between metastasectomy and non-metastasectomy groups using Wilcoxon rank-sum test or Pearson's chi-squared test as appropriate. Follow-up duration was defined as the time interval from the first date of diagnosis of mRCC to the last follow-up or death. Disease progression was defined either as a newly developed metastasis on the postoperative follow-up imaging studies at least 1-month after the surgery for the surgical patients or as the progression according to the RECIST criteria v 1.1 for non-surgical patients. PFS and OS were defined as the time interval from the first date of diagnostic mRCC to the diagnostic date of confirmed disease progression or newly found metastasis and of death. Survival curves were estimated by Kaplan-Meier method, and their differences in PFS and OS between the two groups were tested using the log-rank test. The Cox proportional hazard model was used to evaluate the prognostic significance of metastasectomy in PFS and OS. All factors were included in the multivariable model, after which the backward variable selection method with an elimination criterion of a *p* > 0.05 was applied. As a result, only variables with a *p* < 0.05 were included in the final multivariable model for PFS and OS, respectively. All results were considered statistically significant when two-sided *p* < 0.05. Statistical analysis was performed using SAS 9.4 software (SAS Institute Inc., Cary, NC, USA), and R software, version 3.5.0 (R Project for Statistical Computing).

## Results

### Baseline Characteristics

During a median follow-up time of 143.3 months, median age, PFS, and OS were 57 (10–78) years, 11.6 (1–162.2) months, and 19.9 (1–162.2) months, respectively. Clinical T-staging showed 33.7% of patients had either T3 or T4 stage and that 67.3% had Fuhrman nuclear grade 3–4. Histology results revealed that the prevalence of clear cell type was 87.0%, whereas that of the non-clear cell type was 13.0%. Cytoreductive nephrectomy was performed in 175 (65.3%) patients. Metastases to lung, liver, bone, brain, pancreas, and other sites comprised 216 (79.7%), 64 (23.6%), 117 (43.2%), 51 (19.1%), 12 (4.4%), and 90 (33.0%) cases, respectively. Other information, including baseline clinicopathological characteristics, proportion of prognostic risk groups, and therapeutic modalities, are described in Table 1.

**Table 1:** Baseline characteristics of patients.

		**Number (%)**
Number		273
Age at RCC diagnosis	median (range)	57 (10–78)
Gender	Male	217 (79.5)
	Female	56 (20.5)
Metastatic type	Synchrono metastasis	161 (59.0)
	Metachrono metastasis	112 (41.0)
MSKCC risk group	Favorite	40 (14.7)
	Intermediate	186 (68.1)
	Poor	47 (17.2)
Heng risk group	Favorite	38 (13.9)
	Intermediate	191 (70.0)
	Poor	44 (16.1)
Tyrosine kinase inhibitor	1st	144 (52.9)
	2nd	51 (18.8)
	3rd	22 (8.1)
mTOR inhibitor	1st	5 (1.8)
	2nd	39 (14.3)
	3rd	10 (3.7)
Cytoreductive nephrectomy		175 (64.3)
Metastasectomy		83 (30.4)
Embolization		38 (14.0)
Radiation therapy		114 (41.8)
Tumor	pT1	67 (24.5)
	pT2	60 (22.0)
	pT3	78 (28.6)
	pT4	14 (5.1)
	pTx	54 (19.8)
Fuhrmann nuclear grade	1	10 (5.0)
	2	55 (27.6)
	3	86 (43.2)
	4	48 (24.1)
Histology	clear	208 (87.0)
	Non-clear	31 (13.0)
Sarcomatoid component		15 (5.5)
Number of metastatic lesions	Median (range)	2 (0–8)
Lung metastasis		216 (79.7)
Liver metastasis		64 (23.6)
Bone metastasis		117 (43.2)
Brain metastasis		51 (19.1)
Pancreas metastasis		12 (4.4)
Other metastasis		90 (33.0)

*MSKCC, Memorial Sloan-Kettering Cancer Center; RCC, renal cell carcinoma*.

### Comparison of Results for Metastasectomy and Non-metastasectomy

The baseline comparison between metastasectomy and non-metastasectomy groups is shown in the Table 2. Concerning therapies for primary kidney tumor, cytoreductive nephrectomy was performed in 89.2% (*N* = 74) of cases in the metastasectomy group (*N* = 83) and 53.2% (*N* = 101) in the non-metastasectomy group (*N* = 190) (*P* < 0.001). Radiation therapy was performed in 60.2% (*N* = 50) of cases in the metastasectomy group and 33.7% (*N* = 64) in the non-metastasectomy group (*P* < 0.001). Comparisons between the metastasectomy and non-metastasectomy groups also indicated a significantly younger age, lower clinical T stage (40.5% vs. 60.2%), and higher rate of favorable- and intermediate-risk groups in the metastasectomy group (*p* < 0.05, Table 2). In addition, the metastasectomy group had a significantly higher rate of bone, brain, and pancreas metastases and a lower rate of liver metastasis than that in the non-metastasectomy group (*p* < 0.05).

**Table 2:** Baseline comparison between metastasectomy and non-metastasectomy groups.

		**Total**	**Metastasectomy**		***p*-value**
		**(*n* = 273)**	**No (*n* = 190)**	**Yes (*n* = 83)**	
Age at RCC diagnosis	Median (range)	57 57 (10–78)	58 (10–76)	52 (22–78)	0.0028
**GENDER**					
	Male	217	150 (79.0)	67 (80.7)	0.7382
	Female	56	40 (21.1)	16 (19.3)	
**TUMOR**					
	pT1+pT2	127	77 (40.5)	50 (60.2)	0.0109
	pT3+pT4	92	71 (37.4)	21 (25.3)	
	pTx	54	42 (22.1)	12 (14.5)	
**FUHRMANN NUCLEAR GRADE**					
	1+2	65	41 (32.5)	24 (32.9)	0.961
	3+4	134	85 (67.5)	49 (67.1)	
**HISTOLOGY**					
	Clear	208	142 (87.7)	66 (85.7)	0.6766
	Non-clear	31	20 (12.3)	11 (14.3)	
**MSKCC RISK GROUP**					
	Favorite	40	22 (11.6)	18 (21.7)	0.0039
	Intermediate	186	127 (66.8)	59 (71.1)	
	Poor	47	41 (21.6)	6 (7.2)	
**HENG RISK GROUP**					
	Favorite	38	23 (12.1)	15 (18.1)	0.0008
	Intermediate	191	126 (66.3)	65 (78.3)	
	Poor	44	41 (21.6)	3 (3.6)	
**METASTATIC TYPE**					
	Synchrono metastasis	161	124 (65.3)	37 (44.6)	0.0014
	Metachrono metastasis	112	66 (34.7)	46 (55.4)	
Cytoreductive nephrectomy		175	101 (53.4)	74 (89.2)	<0.001
Embolization		38	28 (14.8)	10 (12.0)	0.5445
Radiation therapy		114	64 (33.7)	50 (60.2)	<0.001
**METASTATIC ORGANS**					
	Lung	216	149 (79.3)	67 (80.7)	0.7819
	Liver	64	52 (27.7)	12 (14.5)	0.0183
	Bone	117	62 (33.0)	55 (66.3)	<0.0001
	Brain	51	28 (15.1)	23 (28.1)	0.0133
	Pancreas	12	5 (2.6)	7 (8.4)	0.0495

*MSKCC, Memorial Sloan-Kettering Cancer Center; OS, overall survival; PFS, progression-free survival; RCC, renal cell carcinoma*.

### Survival Outcomes

**Figure 1:**
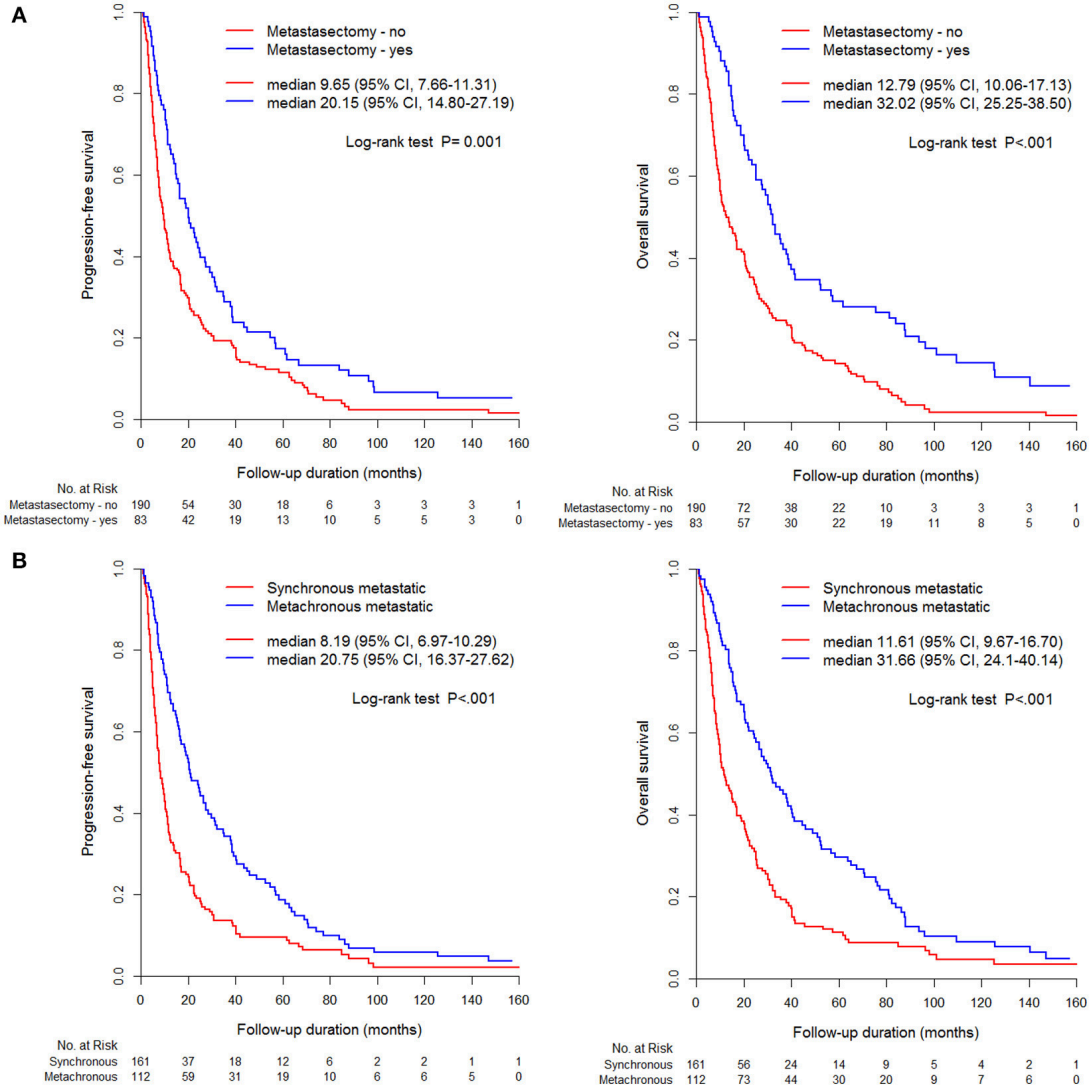
Kaplan-Meier curve for PFS and OS according to **(A)** metastasectomy, **(B)** metastatic type (synchronous/metachronous).

**Figure 2:**
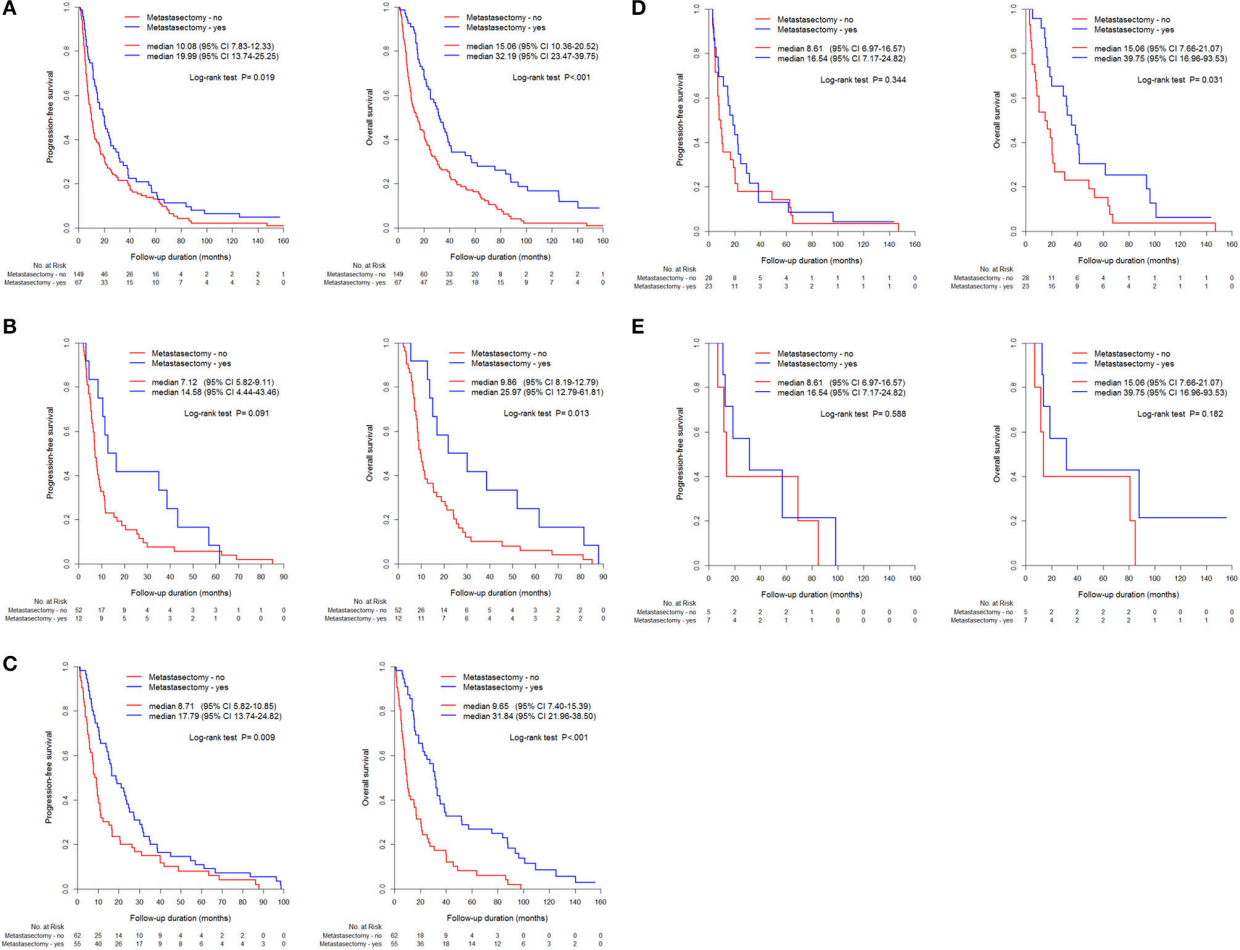
Kaplan-Meier curve for PFS and OS between metastasectomy and non-metastasectomy according to metastatic organs, **(A)** Lung (*N* = 216), **(B)** Liver (*N* = 64), **(C)** Bone (*N* = 117), **(D)** Brain (*N* = 51), **(E)** pancreas (*N* = 12).

The metastasectomy group had significantly better PFS (20.2 vs. 9.7 months) and OS (32.0 vs. 12.8 months) than the non-metastasectomy group (*p* < 0.05, Figure 1A). The synchronous (PFS/OS, 8.2/11.6 months) and metachronous (20.8/31.7 months) metastasectomy showed significant differences in PFS and OS (*p* < 0.05, Figure 1B). Compared with survival following non-metastasectomy, survival for lung and bone metastasectomy had significantly better PFS and OS (*p* < 0.05, Figures 2A,C); liver and brain metastasectomy had only better OS (*p* < 0.05, Figures 2B,D), and pancreas metastasectomy had no significant survival difference (*p* > 0.05, Figure 2E).

**Figure 3:**
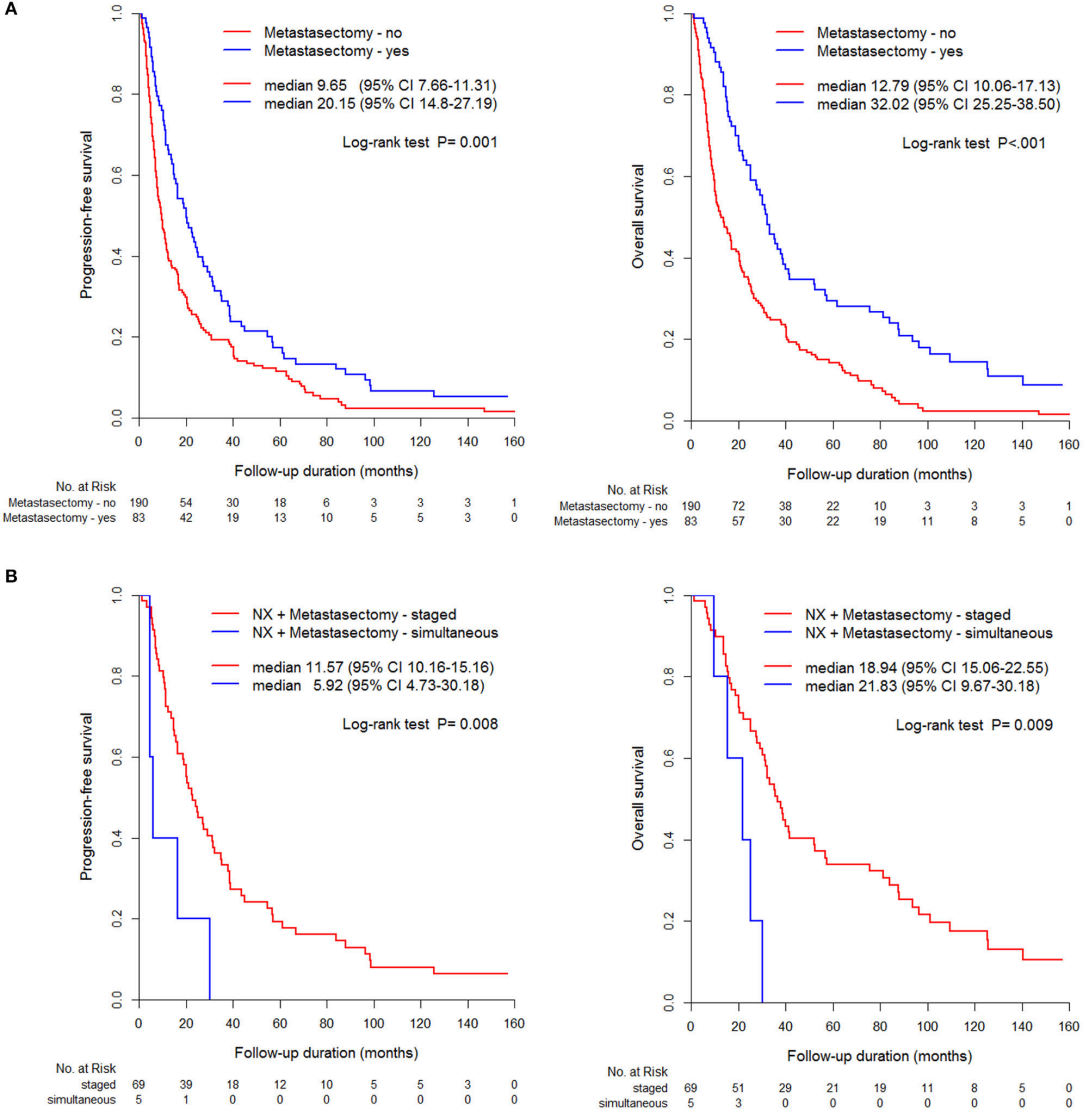
Comparison of PFS and OS between metastasectomy and non-metastasectomy **(A)** without/ **(B)** with nephrectomy.

Comparative survival curves according to surgical methods and timing of cytoreductive nephrectomy and metastasectomy showed that nephrectomy and metastasectomy had the best survival outcome compared to that of other groups, including metastasectomy without nephrectomy and nephrectomy without metastasectomy groups (Figure 3A). Undergoing a staged operation resulted in significantly better PFS and OS than did undergoing simultaneous operation, including nephrectomy and metastasectomy (*p* < 0.05, Figure 3B).

### Multivariable Model of Risk Factors for PFS and OS

As for the prognostic risk factors of PFS and OS, the Cox proportional hazard model showed metastasectomy was a significant independent favorable risk factor for both PFS (hazard ratio [HR] 0.69, 95% confidence interval [95% CI] 0.51–0.93) and OS (HR 0.65 95% CI 0.48–0.88), and cytoreductive nephrectomy was also a significant factor for both PFS (HR 0.39, 95% CI 0.28–0.53) and OS (HR 0.47, 95% CI 0.34–0.65) (*p* < 0.05). Using Heng risk criteria, only OS was statistically significant for intermediate risk (HR 1.66, 95% CI 1.12–2.44) and poor risk (HR 3.21, 95% CI 1.9–5.43) groups (*p* < 0.05). Non-clear cell histology (HR 0.61, 95% CI 0.40–0.93) and radiation therapy (HR1.61, 95% CI 1.22–2.12) were significant factors only for PFS (*p* < 0.05, Table 3).

**Table 3:** Univariable and multivariable using the Cox proportional hazard model.

		**PFS (*****n*** **=** **273, event** **=** **256)**				**OS (*****n*** **=** **273, event** **=** **245)**			
		**Univariable**		**Multivariable**		**Univariable**		**Multivariable**	
		**Hazard ratio**	***p*-value**	**Hazard ratio**	***p*-value**	**Hazard ratio**	***p*-value**	**Hazard ratio**	***p*-value**
**METASTASECTOMY**									
	No	1 (ref)		1 (ref)		1 (ref)		1 (ref)	
	Yes	0.64 (0.49–0.84)	0.0012	0.69 (0.51–0.93)	0.0164	0.51 (0.38–0.67)	<0.0001	0.65 (0.48–0.88)	0.0047
Age at RCC diagnosis	Median (range)	1.01 (1.00–1.02)	0.1364			1.01 (1.00–1.02)	0.0733		
**GENDER**									
	Male	1 (ref)				1 (ref)			
	Female	1.28 (0.95–1.73)	0.1044			1.24 (0.91–1.68)	0.1681		
**TUMOR**									
	pT1 + pT2	1 (ref)	(0.1742)			1 (ref)	(0.5879)		
	pT3 + pT4	1.28 (0.97–1.68)	0.0841			1.13 (0.85–1.49)	0.4125		
	pTx	0.99 (0.70–1.38)	0.9296			1.17 (0.83–1.65)	0.3822		
**FUHRMANN NUCLEAR GRADE**									
	1 + 2	1 (ref)				1 (ref)			
	3 + 4	1.20 (0.88–1.64)	0.2593			1.10 (0.80–1.53)	0.5493		
**HISTOLOGY**									
	Clear	1 (ref)		1 (ref)		1 (ref)			
	Non-clear	0.53 (0.35–0.79)	0.0021	0.61 (0.40–0.93)	0.0205	0.65 (0.44–0.98)	0.0392		
**MSKCC RISK GROUP**									
	Favorite	1 (ref)	(<0.0001)			1 (ref)	(<0.0001)		
	Intermediate	1.60 (1.13–2.28)	0.0089			1.88 (1.30–2.72)	0.0008		
	Poor	2.78 (1.79–4.32)	<0.0001			3.60 (2.29–5.66)	<0.0001		
**HENG RISK GROUP**									
	Favorite	1 (ref)	(<0.0001)			1 (ref)	(<0.0001)	1 (ref)	(<0.0001)
	Intermediate	1.67 (1.16–2.41)	0.0058			1.90 (1.30–2.78)	0.0009	1.66 (1.12–2.44)	0.0109
	Poor	4.14 (2.62–6.55)	<0.0001			5.84 (3.62–9.40)	<0.0001	3.21 (1.90–5.43)	<0.0001
**METASTATIC TYPE**									
	Synchrono metastasis	1 (ref)				1 (ref)			
	Metachrono metastasis	0.55 (0.43-0.71)	<0.0001			0.54 (0.42–0.71)	<0.0001		
**CYTOREDUCTIVE NEPHRECTOMY**									
	No	1 (ref)		1 (ref)		1 (ref)		1 (ref)	
	Yes	0.36 (0.27–0.47)	<0.0001	0.39 (0.28–0.53)	<0.0001	0.32 (0.24–0.42)	<0.0001	0.47 (0.34–0.65)	<0.0001
**EMBOLIZATION**									
	No	1 (ref)				1 (ref)			
	Yes	1.53 (1.07–2.19)	0.0191			1.57 (1.09–2.26)	0.0147		
**RADIATION THERAPY**									
	No	1 (ref)		1 (ref)		1 (ref)			
	Yes	1.22 (0.95–1.56)	0.1221	1.61 (1.22–2.12)	0.0007	0.97 (0.75–1.25)	0.7895		

*MSKCC, Memorial Sloan-Kettering Cancer Center; OS, overall survival; PFS, progression-free survival; RCC, renal cell carcinoma; ref, reference variable*.

### Prognostic Impact on Survival Based on the Metastatic Organs

In the influential hazard analyses of each metastatic organ that underwent metastasectomy compared to non-metastasectomy, liver metastasis was the only significant unfavorable risk factor for both PFS (HR 1.67, 95% CI 1.25–2.22) and OS (HR 1.74, 95% CI 1.30–2.33) (*p* < 0.001, Table 4). The worst combination of organ metastasis was liver and lung, which resulted in significantly unfavorable PFS (HR 2.22, 95% CI 1.07–4.58) and OS (HR 2.62, 95% CI 1.23–5.58) compared to those of other combinations of metastatic organs (*p* < 0.05, Table 5).

**Table 4:** Overall survival and progression-free survival according to metastatic lesions.

		**Total**	**PFS**			**OS**		
			**Event (%)**	**HR (95% CI)**	***p*-value**	**Event (%)**	**HR (95% CI)**	***p*-value**
Lung metastatic	No	55	50 (90.9)	1 (ref)		49 (89.1)	1 (ref)	
	Yes	216	204 (94.4)	0.99 (0.72–1.34)	0.9245	194 (89.8)	0.85 (0.62–1.17)	0.3202
Liver metastatic	No	207	190 (91.8)	1 (ref)		180 (87.0)	1 (ref)	
	Yes	64	64 (100.0)	1.67 (1.25-2.22)	0.0005	63 (98.4)	1.74 (1.30-2.33)	0.0002
Bone metastatic	No	154	139 (90.3)	1 (ref)		134 (87.0)	1 (ref)	
	Yes	117	115 (98.3)	1.23 (0.96–1.58)	0.0987	109 (93.2)	1.11 (0.86–1.43)	0.4381
Brain metastatic	No	216	201 (93.1)	1 (ref)		193 (89.4)	1 (ref)	
	Yes	51	50 (98.0)	1.07 (0.79–1.46)	0.6684	47 (92.2)	0.93 (0.68–1.28)	0.6561
Pancreas metastatic	No	261	245 (93.9)	1 (ref)		235 (90.0)	1 (ref)	
	Yes	12	11 (91.7)	0.62 (0.34–1.14)	0.121	10 (83.3)	0.66 (0.35–1.25)	0.2042

*HR, hazard ration; OS, overall survival; PFS, progression-free survival; ref, reference variable*.

**Table 5:** The prognostic changes of progression-free survival and overall survival by additional organ metastasis in patients with liver metastasis.

	**Total**	**PFS**			**OS**		
		**Event (%)**	**HR (95% CI)**	***p*-value**	**Event (%)**	**HR (95% CI)**	***p*-value**
No metastatic	14	11 (78.6)	1 (ref)		10 (71.4)	1 (ref)	
Only Liver metastatic	10	10 (100.0)	1.59 (0.67–3.78)	0.2971	10 (100.0)	2.09 (0.86–5.1)	0.1055
(Liver + Lung) metastatic	23	23 (100.0)	2.22 (1.07–4.58)	0.0320	23 (100.0)	2.62 (1.23–5.58)	0.0126
(Liver + Lung + Bone) metastatic	14	14 (100.0)	1.95 (0.87–4.33)	0.1030	14 (100.0)	2.06 (0.91–4.65)	0.0834
(Liver + Lung + Bone + Brain) metastatic	2	2 (100.0)	3.36 (0.72–15.61)	0.1221	1 (50.0)	2.63 (0.33–21.09)	0.3625

*HR, hazard ration; OS, overall survival; PFS, progression-free survival; ref, reference variable*.

## Discussion

Over the past few decades, despite a lack of randomized clinical trials concerning metastasectomy for mRCC, metastasectomy has been known for its clinically favorable benefit beyond palliation, with 20% 5-year OS and survival time ranging from 10 to 20 months (16). This favorable outcome has been suggested to be the effect of resection of the primary tumor for most patients when they underwent metastasectomy with nephrectomy; (3, 17) in addition, new systemic targeted therapy elicited significant beneficiary gains in survival (18). This study not only focused on the significant survival efficacy of metastasectomy during targeted therapy in mRCC, but also found several significant favorable risk factors for survival, similar to those of previous studies (19, 20). The present study performed additional subgroup analyses among metastasectomy and non-metastasectomy patients to determine risk factors for PFS and OS and that group stratification was the only significant risk factor between the two groups for both PFS and OS.

The efficacy of metastasectomy should be discussed in various situations wherein the effect of targeted therapy for metastectomized mRCC might differ from that of macroscopic tumor burden without surgical resection; moreover, the role of postoperative targeted therapy after metastasectomy and cytoreductive nephrectomy in this setting has not been well-understood. To better examine the effects of targeted therapy, several randomized controlled studies (NCT01216371, NCT01575548, NCT02855203, NCT03024996) are enrolling prospective, multicenter cohorts of patients undergoing metastasectomy; these studies will improve understanding of outcomes following metastasectomy, including the utility of postoperative systemic therapy (21). However, until these results are realized, understanding the benefit of metastasectomy depends on retrospective studies and systemic reviews, including the present study, as well as other previously reported studies from other cancer types that illustrate the significant role of metastasectomy (8, 9, 12, 21, 22).

Ost et al. (23) reported the results of a randomized clinical trial in metastatic prostate cancer concerning the effect of consolidative therapy (metastasis-directed therapy [MDT]) compared to the effect of surveillance; effects examined included the prevention of additional metastatic spread and improvements in survival. The MDT group had significantly longer symptom-free time, local progression-free time, and hormone therapy-free time compared to that of the surveillance group, although the MDT group also progressed to polymetastasis MDT. Future trials may show that this concept of MDT therapy might improve PFS and OS in addition to a temporary systemic drug; furthermore, MDT might be used for mRCC with metastasectomy in conjunction with systemic targeted therapy to prolong symptom-free time, local-progression-free time, polymetastasis-free time, or OS. Because metastasis-free survival is a surrogate for OS, this synergistic approach might improve the therapeutic outcome by eradicating microscopic disease.

An NSCLC study by Gomez et al. (24) reported the efficacy of metastasectomy as a consolidative therapy for metastatic disease in a stage 4 study. In 74 eligible patients treated with either local consolidative therapy (chemoradiation or complete metastasectomy) with or without maintenance treatment or observation, the consolidative therapy group had longer PFS and longer time to the appearance of a new lesion (11.9 months vs. 5.7 months, *p* = 0.0497); this suggests that local consolidative treatment could alter the natural history of the disease, either by limiting the potential for later spread or possibly by altering systemic anticancer immune responses, thus facilitating longer control of subclinical disease. In a systematic review by Dabestani et al. (9) concerning the therapeutic effect for mRCC with regard to metastases to parenchymal organs, metastasectomy with complete resection provided a significant survival benefit compared to that of either incomplete metastasectomy or no metastasectomy. The evidence favored local treatment (metastasectomy or radiation therapy, after a combination of targeted treatment and local treatment) in terms of symptom control, long-term complete response rate, delay of return to systemic treatment and associated toxicity, and PFS (9, 25).

Metastasectomy of different organs also differentially influences survival in mRCC (8, 9, 25). In the present study, metastasectomy of lung, bone, brain, and pancreas showed significant prognostic benefit for survival in mRCC, whereas in a systematic review, metastasectomy of liver and lymph nodes failed to show a benefit or lacked sufficient evidence to support a benefit in mRCC. Liver metastasis in this study also showed a poor prognosticator of survival, with a median OS of 7.4 months and a similar diagnostic prevalence of liver metastasis in mRCC (18.8%) as that previously reported (20%) (26). The current study showed that liver metastasectomy resulted in the poorest PFS (HR 1.67) and OS (HR 1.74), especially in combination with lung metastasectomy (HR 2.22) (*p* < 0.05, Tables 3, 4); moreover, liver metastasectomy resulted in a non-significant improvement in survival compared to that in non-metastasectomy (Figure 2). Although solitary lung metastasectomy and complete removal of multiple lung metastases were significant favorable prognostic factors for mRCC (10), a significantly worse prognosis was observed in patients who underwent metastasectomy simultaneously for both liver and lung metastases (20–22).

Because solitary metastases comprise just 2–4% of liver metastases, poor survival following liver metastasectomy may be a result of multiple metastases due to direct seeding of metastatic tumor cells via the dual hepatic blood supply to a hypervascular pulmonary organ; alternatively, the hepatic blood supply may initiate systemic seeding to other organs. This is in accordance with the hematogenous spread pattern of RCC (27). Additionally, liver metastasis responds poorly to systemic therapy, with a 15% objective response rate to immunochemotherapy, including high dose interleukin-2 (10, 26). This can be partly explained by the high morbidity (up to 33%) and mortality (<5%) rates following liver resection, including that performed owing to hepatic malfunction. Immune function in the tumor microenvironment may also explain the poor outcome following liver metastasectomy, wherein the metastatic tumor cells are resistant to the hyperimmune and detoxifying hepatic system; this may be due to an aggressive genetic background that resists systemic therapy, even after the decreased tumor burden from metastasectomy (28, 29).

Brain metastasis occurs at an incidence of about 8% in synchronous mRCC (30, 31) and 2.4% in metachronous mRCC (32); the current findings show that PFS after brain metastasectomy showed no significant surgical benefit, despite the non-significantly higher median PFS compared with that of non-metastasectomy. The lack of significance may be because routine brain imaging studies have not typically been performed for mRCC evaluation until neurologic symptoms develop; therefore, early detection of brain metastasis, which likely would decrease morbidity, was not obtained, thus advanced tumor burdens did not significantly differentiate PFS.

Contrary to previous studies showing that metachronous type with a significantly longer disease-free interval (>1 year) correlated with a favorable survival (20, 32), in the present study, metastatic type and disease-free duration were not significant prognostic factors for either PFS or OS in view of metastasectomy. This may be because of the higher aggressiveness of the recurrent and metastasized tumors compared to that of the newly diagnosed synchronous metastatic tumors due to intra-tumor heterogeneity and heterogeneous genetic differences between primary and metastatic RCC (33, 34). However, similar to a previous study (8), both prognostic risk stratifications (Heng or MSKCC criteria) were indicative of unfavorable survival risk (Table 3), Moreover, non-clear histology is also a favorable risk factor for PFS in mRCC (HR 0.61, *p* = 0.022, Table 3) because there is no efficacious current systemic therapy for non-clear histology; furthermore, this study showed that a surgical decrease in tumor burden following metastasectomy is an efficacious therapy for non-clear cell mRCC.

Some limitations exist in this study, including the retrospective, single-center design; the low number of cases; the lack of consideration of the number of metastatic lesions within the metastatic organs, and the sizes or total sum of the metastatic lesions, representing the overall tumor burden; and the derivation of results over an extended period. Baseline general performance states and underlying disease were not considered thoroughly; these include prognostic risk stratification; other adjuvant therapeutic modalities; other metastatic sites such as adrenal gland, contralateral kidney, and lymph nodes; and systemic therapeutic modalities such as cytokine or targeted agents. To evaluate further the efficacy of metastasectomy requires additional larger, multicenter studies or propensity-score matching to adjust for inherent selection biases regarding patients underwent surgery compared to non-metastasectomy patients.

## Conclusion

The study showed a significantly beneficiary role of metastasectomy according to various metastatic states in survival gains with regards to mRCC and found that liver metastasis was the most unfavorable factor in metastasectomy in mRCC.

## Ethics Statement

Following approval of this retrospective study by the Institutional Review Board of the National Cancer Center (IRB No. NCC 2015-0212), the IRB approved exemption from the written consent procedure. All study protocols were performed in accordance with the tenets of the ethical guidelines and regulations of the World Medical Association Declaration of Helsinki-Ethical Principles for Medical Research Involving Human Subjects.

## Author Contributions

SK: conceptualization, data curation, investigation, methodology, project administration, supervision and writing—original draft preparation. WP: conceptualization, data curation, investigation, methodology, supervision and writing—original draft preparation. BP: conceptualization, data curation, formal analysis, investigation, methodology, project administration, supervision and writing—original draft preparation. JC: conceptualization, data curation, investigation, methodology, project administration, supervision, funding acquisition and writing—original draft preparation. SP: Data Curation, Investigation - Revised Draft Preparation.

### Conflict of Interest Statement

The authors declare that the research was conducted in the absence of any commercial or financial relationships that could be construed as a potential conflict of interest.

## Supplementary Material

Supplementary Figure 1 Flow diagram of detailed information regarding the study population.
