# Supplementary figures and images for: A Retrospective Analysis of the Impact of Metastasectomy on Prognostic Survival According to Metastatic Organs in Patients With Metastatic Renal Cell Carcinoma
Source: Front Oncol. 2019 May 22;9:413. doi: 10.3389/fonc.2019.00413 (PMC6538800; doi:10.3389/fonc.2019.00413)

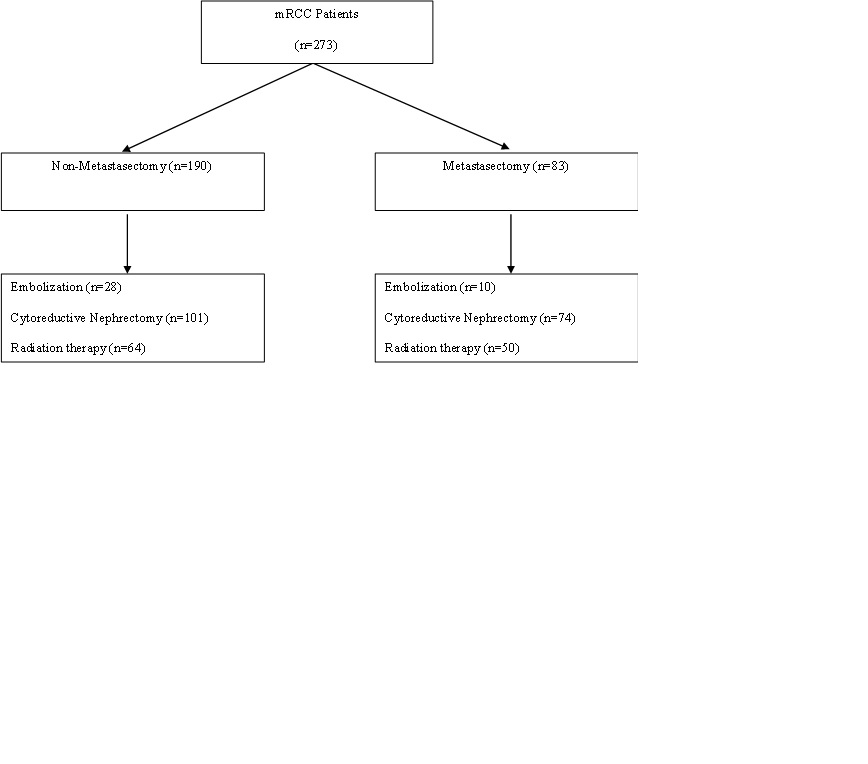

Supplement: Supplementary Figure 1 — Flow diagram of detailed information regarding the study population. [file Image_1.JPEG]
